# Early identification of bovine pregnancy status and embryonic mortality^†^

**DOI:** 10.1093/biolre/ioaf066

**Published:** 2025-03-28

**Authors:** Jeanette V Bishop, Aydin Guzeloglu, Tom Scheller, Joshua J Docheff, Carolina L Gonzalez-Berrios, Hana Van Campen, Terry M Nett, Abigail L Zezeski, Thomas W Geary, William W Thatcher, Thomas R Hansen

**Affiliations:** Animal Reproduction and Biotechnology Laboratory, Department of Biomedical Sciences, Colorado State University, Fort Collins, CO, USA; Animal Reproduction and Biotechnology Laboratory, Department of Biomedical Sciences, Colorado State University, Fort Collins, CO, USA; PTSAgLLC, Fort Collins, CO, USA; Blue Sky Dairy, Mead, CO, USA; Animal Reproduction and Biotechnology Laboratory, Department of Biomedical Sciences, Colorado State University, Fort Collins, CO, USA; Animal Reproduction and Biotechnology Laboratory, Department of Biomedical Sciences, Colorado State University, Fort Collins, CO, USA; Animal Reproduction and Biotechnology Laboratory, Department of Biomedical Sciences, Colorado State University, Fort Collins, CO, USA; United States Department of Agriculture, Agricultural Research Service, Fort Keogh Livestock and Range Research Laboratory, Miles City, MT, USA; United States Department of Agriculture, Agricultural Research Service, Fort Keogh Livestock and Range Research Laboratory, Miles City, MT, USA; University of Florida, Department of Animal Sciences, Gainesville, FL, USA; Animal Reproduction and Biotechnology Laboratory, Department of Biomedical Sciences, Colorado State University, Fort Collins, CO, USA

**Keywords:** bovine, pregnancy, conceptus, interferon-tau, diagnostic

## Abstract

Bovine interferon-tau (bIFNT) is produced by the trophectoderm cells in the bovine conceptus as early as Day 12 following fertilization. It was hypothesized that IFNT detection in blood, milk, and/or cervical secretions could be used to diagnose pregnancy in lactating cows. Recombinant bovine (rb) IFNT was generated to produce goat and rabbit anti-rbIFNT polyclonal antibodies, and an enzyme-linked immunosorbent assay (ELISA) was developed for bIFNT using these reagents. The IFNT ELISA did not cross-react with other type I or II IFNs and had a limit of detection of 50–100 pg/ml. The IFNT ELISA detected IFNT in external ostium (os) cervical swabs from Days 15 to 25 post-AI, but did not detect IFNT in serum, plasma, or milk. The time for most accurately detecting IFNT in cervical fluid was Days 16–19 after AI. A custom bovine swab device used to collect cervical secretions reduced false-negative rates to 5.5% (94.5% sensitivity) in dairy cows on Day 17 and 0% to 3.4% (100% and 96.6% sensitivity) in beef cows on Days 18 or 16, respectively. In summary, the detection of IFNT in cervical fluid by ELISA provides an accurate indication of pregnancy status in lactating dairy cows. Early identification of the non-pregnant cow allows re-insemination on Day 21 compared to waiting until ultrasound (US) on Day ~32–39. In addition, the detection of IFNT on Day 17 followed by the loss of pregnancy detected by US on Day 32 provides a novel research tool for studying pregnancy loss caused by embryonic mortality.

## Teaser

A rapid test has been developed to identify bovine pregnancy status 17 days after insemination, allowing re-synchronization of estrous cycles and re-insemination of open cows to achieve pregnancy 2–3 weeks earlier compared to current diagnostics.

## Graphical Abstract

**Graphical Abstract:**
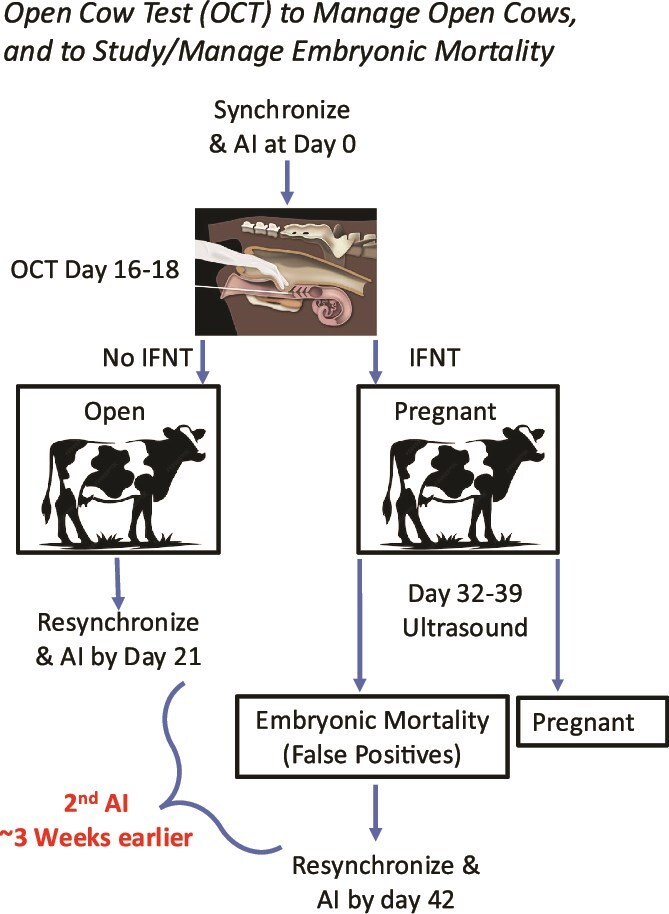

## Introduction

Lactating dairy cows in the USA have first-service pregnancy rates of 31%–60% following artificial insemination (AI) at estrus or timed AI using estrous synchronization schemes (reviewed in [1]). The current gold standard method for pregnancy diagnosis is transrectally- guided ultrasound (US) on Day 32 or later after AI [2, 3], and 40%–69% of cows are diagnosed as not pregnant (open) on Day >32. Because of this low first-service conception rate, dairy cows often undergo multiple rounds of AI each year before becoming pregnant. The later in lactation that pregnancy occurs each year, the lower the annual milk production and the lesser the financial return in subsequent years.

Additional methods for pregnancy diagnosis include palpation per rectum 28–35 days following AI [4]. However, this method is associated with an increased risk of fetal damage or losses of pregnancy [5, 6]. Pregnancy diagnosis by transrectally-guided US [7, 8] is accurate as early as Day 27 after AI when performed by a skilled technician [9–12] although most practitioners use US for pregnancy diagnosis on Days 32–39 following AI. Another accepted method of pregnancy diagnosis is based on the detection of a pregnancy-specific protein called Pregnancy specific protein B (PSPB) or pregnancy-associated glycoprotein (PAG1) by radioimmunoassay in serum/plasma on Day 32 post-AI [13–17]. However, PAG1 is not detectable prior to the time when US can be used for pregnancy diagnosis. In comparison, the type I IFNT is produced by the trophectoderm cells in the bovine conceptus as early as Day 12 following fertilization, and *IFNT* mRNA is expressed specifically by the mononuclear trophectoderm cells of the conceptus from Day 15 to 25 with peak levels on Days 17–19 of pregnancy [18]. It has also been reported that IFNT is not induced [19] or is very weakly induced by the virus [20], although these are in vitro studies. In vivo, it has also been determined that IFNT is not induced by bacterial infusion into the bovine uterus (endometritis) during early pregnancy [21, 22]. For this reason, the present experiments were focused on developing a rapid and accurate ELISA for bovine IFNT to identify the open lactating cow on Days 16–19 using an open cow test (OCT) based on the absence of IFNT.

An early OCT may also have utility in beef females. Only 60%–70% of successful inseminations result in a viable pregnancy in beef cows [23]. Early loss of embryos is also a problem in these cattle. However, unlike dairy cows, three opportunities to conceive in beef cows result in ~95% pregnancy rates. Beef heifers are typically synchronized one or two times for AI and then are placed on pasture with bulls. Using the OCT approach on Day 17 will allow more rapid resynchronization prior to placing females on pasture with cleanup bulls.

By identifying those cows with detectable IFNT concentrations on Days 16–17 followed by the loss of embryos determined by US, the IFNT ELISA may be used in the future to track embryonic mortality. This tool may enhance our understanding of early pregnancy failures with the goal of developing methods to re-synchronize estrus and reprogram the uterine environment for insemination.

## Materials and methods

### Generation of recombinant glycosylated bovine interferon-tau

The full-length 1.1 kb cDNA clone encoding bIFNT—called bovine trophoblast protein (BTP509)—was isolated, subcloned into pUC13, and sequenced [24, 25] at the University of Missouri (Dr. Michael Roberts), which was then transferred to Colorado State University (Supplementary Figure S1). The BTP509 clone was modified by the addition of nucleotides encoding a C-terminal 6× HIS tail that aided in purification. Glycosylated recombinant bovine (rb) IFNT (rbIFNT) was generated in human embryo kidney (HEK) cells in collaboration with a biopharma company and tested for antiviral activity by our laboratory as described [26]. Briefly, the IFNT cDNA clone was codon optimized for expression in mammalian cells by ATUM Bios (Newark, CA). This modified BTP509 clone was subcloned into their pD2529 plasmid using the Electra cloning system (Supplementary Figure S1). The Electra system uses the type IIS restriction enzyme Streptomyces aureus P I (*SapI*), which recognizes a 7-bp non-palindromic recognition sequence and leaves a 3-bp 5′ overhang after digestion. IFNT was expressed transiently in suspension Freestyle 293-F cells, which were maintained in Freestyle 293 (derived from HEK cells) expression medium (Gibco Scientific through Thermo Fisher Scientific, Waltham, MA) between 0.15 and ~3 × 10^6^ cells/ml. The cells were diluted following the manufacturer’s instructions for maintenance passaging and transfection day. The diluted cells were transfected as described in the protocol using reagents sourced from Polysciences (Warrington, PA) for polyethyleneimine Max transfection reagent. After 7 days of incubation, the cultures were harvested and the clarified supernatant was purified. The identity of glycosylated recombinant rbIFNT protein was confirmed by western blot using anti-rbIFNT antibody provided by Dr. Michael Roberts (University of Missouri) and mass spectroscopy amino acid sequencing of several rbIFNT peptides by the Colorado State University Proteomics Core facility.

### Generation of polyclonal rabbit and goat antibodies against recombinant bovine IFNT

Recombinant bovine IFNT antigen (from HEK transient culture) was submitted to Maine Biologicals Services (currently BBI Solutions, Portland, ME) for the production of antisera in rabbits. Rabbits were immunized initially with 250 μg of rbIFNT in Complete Freund’s Adjuvant and boosted with 125 μg of rbIFNT in Incomplete Freund’s Adjuvant every 3 weeks. All immunizations were subcutaneous. Recombinant bIFNT antigen (from HEK transient culture) was submitted to Zoetis VMRD Richland Farms (Richland, MI) for the production of antisera in goats. Goats were immunized initially with 150 μg of protein in a Zoetis Adjuvant and boosted with 150 μg of protein in Zoetis Adjuvant. All immunizations were subcutaneous. Several booster immunizations and blood samples from the rabbits and goats over 1 year were tested for the ability to detect rbIFNT using western blot, radioimmunoassay (not shown), and sandwich ELISA (described herein). Monoclonal antibodies were also generated, but unfortunately, the hybridomas died.

### The recombinant bovine IFNT sandwich enzyme-linked immunosorbent assay protocol

Goat and rabbit anti-bIFNT polyclonal sera were purified over Protein A/G GraviTrap equilibrated columns (Millipore, Sigma, Burlington, MA) by mixing 2 ml of serum with an equal volume of binding buffer as specified. Protein concentration of the fractions was determined using the Pierce bicinchoninic assay (Thermo Fisher Scientific, Waltham, MA), and fractions containing antibodies were pooled. The Goat 51 bIFNT antibody serum was further concentrated using an Amicon 100 k MNWL Ultra 0.5 ml centrifugal spin column (Merck Millipore, Rockville, MD). Multiple matrices of capture and detector antibody dilutions at different concentrations of rbIFNT were tested in ELISAs over one year of booster antibody production were performed to determine the best polyclonal antibody for capture and detection in context of rbIFNT concentrations tested for the ELISA standard curve.

Goat #51 anti-bIFNT polyclonal antibody was added to 96-well plates (Greiner Bio-One, Frickenhausen, Germany) as the capture antibody at 3.5 μg/ml in 0.2 M sodium carbonate/bicarbonate (pH 9.4) and incubated for 1 h at 25°C. The plates were washed three times using ELISA Wash Buffer (0.025 M Tris, 0.15 M Sodium Chloride pH 7.2, 0.05% Tween 20). The plates were blocked (300 μl/well) for 1 h at 25°C in 2% BSA Fraction V (EMD Millipore, Darmstadt, Germany) using ELISA Wash Buffer. To generate a standard curve, rbIFNT protein was diluted to yield a linear curve in 10% steer serum (serum diluted in 1× Phosphate-Buffered Saline [PBS]) at 0, 40, 100, 200, 300, 400, 500, and 600 pg/ml. All standards, samples, buffer reagents, and quality controls were added to wells in duplicate 50 μl aliquots except for 2% blocking buffer at 300 μl.

Each undiluted swab sample (vulva, vagina, and cervix) was collected in either 0.3 or 1 ml of 1× PBS and was added to a 96-well titer plate in duplicate, and the plates were incubated at 37°C for 1 h. The wells were washed and then 9.6 μg/ml of detector antibody (rabbit 5670 polyclonal antibody) conjugated to biotin (FluoReporter Mini-biotin-XX Protein Labeling Kit #F6347, Life Technologies, Eugene, OR) was added and incubated for 1 h at 25°C. The plates were washed, SA-HRP (#DY998 R&D Systems, Minneapolis, MN) was added at a 1:200 dilution, and the plates were incubated for 30 min at 25°C and then washed six times. An equal mixture of 3,3′,5,5′ tetramethylbenzidine substrates A and B (#42110, BioLegend, San Diego, CA) was added, followed by incubation for 4–6 min, with termination of the assay using 1.6 M H_2_SO_4_. The plates were read at 450 nM on a BioTech Synergy 2 reader (Winooski, Vermont).

Approximately 1 l of steer blood was collected from a single steer. Sera were separated by centrifugation at 400 × *g* for 30 min at 4°C. The serum phases were pooled at the lab and aliquoted for storage at ^−^20°C for use in dilution of the standards, samples, and controls. All standards, samples, background, and quality controls were added to duplicate wells at 50 μl. Quality control rbIFNT standards were diluted in 10% steer serum (500, 100, and 20 pg/ml) and were added to each plate to determine the coefficients of variation.

The average rbIFNT standard absorbance at OD450 nM was plotted to generate a standard curve, and the rbIFNT concentrations were reported in pg/ml. The coefficient of variation (%) was calculated as the standard deviation between rbIFNT OD450 absorbance wells/means × 100. In these studies, the rbIFNT ELISA had an average stringent limit of detection (LOD) of 54 pg/ml (2× the OD450 of serum control). The IFNT ELISA was tested for antigen specificity to bIFNT. Other type 1 IFN proteins such as IFNα, IFNβ, and IFNγ (Kingfisher Biotech, St. Paul, MN), and IFNω (CUSABIOTECH, Wuhan, China) were tested on the same 96-well plates with rbIFNT protein at concentrations of 1000, 500, 100, 50, 10, 5, and 1 pg/ml.

### Accuracy of the bIFNT enzyme-linked immunosorbent assay to identify open cows compared to ultrasound diagnosis on Day 32

The accuracy of OCT to identify open (negative) and pregnant (positive) cows compared to US diagnosis was calculated for each assay. See accuracy categories listed below:

**Sensitivity (true positive [TP] rate)**: Correct ELISA pregnant = # Pregnant by both ELISA & US/# pregnant by US.

**Specificity (true negative [TN] rate):** Correct ELISA open = # Open by both ELISA & US/# open by US.

**False positive (FP) rate:** Pregnant by ELISA but found to be open by US = # ELISA false pregnant/# open by US.

**False negative (FN) rate:** Open by ELISA but pregnant by US = # ELISA false open/# pregnant by US.

**Negative predictive value (NPV):** Proportion of non-pregnant animals that were called non-pregnant = # open by both ELISA and US/open by ELISA.

The FP rates are expected because conceptuses that are present on Day 17 often die (up to 40%) prior to US diagnosis [1, 27]. The two primary factors that are of concern for the OCT are FN and specificity estimates. Furthermore, the NPV is of great utility when deciding on re-breeding strategies to avoid aborting any pregnant cows if rebreeding strategies are using prostaglandin F_2α_ (PGF). If the FN value is significant (>10%), then the OCT may still be useful, but strategies for rebreeding that do not include PGF need to be considered.

### Ethics: Experimental animals

The studies with lactating dairy cows (Holstein and Jersey cows) were reviewed and approved by the Colorado State University Institutional Animal Care and Use Committee (protocol numbers: 1895, 1043, and 445). All protocols used at USDA-ARS were approved by the Institutional Animal Care and Use Committee (approval protocol #61322-1 and 61322-2). Blood, milk, and swab samples were collected in collaboration with two nearby commercial dairies. The pregnancy status for each cow based on OCT was exchanged electronically at the same time on the same day (blinded) with each dairy cooperator and for beef cattle to Dr. Geary at USDA-ARS after US analysis on Day 32+.

### Animals and estrous synchronization

Colorado Dairy A is located in the southeast of Fort Collins, Colorado, and provided access to their lactating dairy cows (primarily Holstein, *n* = 34, Day 17 cows) for these studies. A modification of the G6G protocol (inject PGF followed two days later with an injection of Gonadotropin-Releasing Hormone (GnRH) and then start the Ovsynch protocol 6 days later) [28] was used to synchronize ovulations. The cows were inseminated 16 h after the last GnRH injection of the Ovsynch protocol. The pregnancy of the cows was checked by transrectal US at Day 32. This dairy typically has pregnancy rates of 32%–40% at Day 32 based on US. Colorado Dairy B is located in the northeast of Fort Collins, CO. Lactating Holstein dairy cows (*n* = 323 and 99 cows) were synchronized using a Double-Ovsynch program [29] with a GnRH injection followed by an injection of PGF 7 days later on or around Day 53 postpartum, followed by GnRH 3 days later and then initiation of the Ovsynch-timed AI protocol 7 days later. The target of the first AI service was around Day 80. This dairy typically has pregnancy rates of 43% at a Day 39 post-breeding US.

Beef cattle (Hereford × Angus, and Red Angus × Charolais × Tarentaise composite cows) collected through the USDA-ARS in Miles City, Montana, were synchronized using the 5-day CO-Synch + CIDR protocol with breeding by estrus or timed AI and then cervical fluid was collected using the equine swab (ES) device as described. Heifers (*n* = 6) not inseminated and external os cervical swab samples were collected on Day 19 to serve as TN (non-pregnant) controls. Other heifers were inseminated following the detection of estrus and then swabbed on Days 18 (*n* = 5), 19 (*n* = 28), or 20 (*n* = 68). Beef cows (*n* = 135) were inseminated and swabbed on Day 18. Furthermore, beef cows (*n* = 126) that received a Day 7 frozen/thawed embryo following embryo transfer on Day 7 were swabbed on Day 18. Subsequent herds of beef cattle were inseminated and then swabbed with the new improved bovine swab (BS) device on Day 16 (*n* = 70) or 18 (*n* = 66) after AI.

### Collection of milk, serum, plasma, vulva, vagina, and cervical os samples (Colorado Dairy A)

#### Milk

Milk (50 ml) was collected on Days 0, 17, 19, and 21 following AI by the dairy from 24 dairy cows and then transported to the laboratory on ice. The whole milk was centrifuged at 400 × *g* for 30 min and the fat layer was removed. For the standard curve, rbIFNT was spiked into the skimmed milk to generate a serially diluted rbIFNT standard curve. All experimental skim milk samples were analyzed concurrently with the serially diluted rbIFNT standard curve in skimmed milk. Blood from tail veins (~8 ml) from each cow was collected in purple top vacutainers (Thermo Fisher Scientific, Waltham, MA) for plasma after centrifugation at 400 × *g* for 30 min, which was stored at −20°C. Blood was collected from the same cows as for milk on Days 0, 17, 19, and 21 following AI. Plasma was collected after centrifugation of blood at 400 × *g* for 30 min and stored at −20°C. The rbIFNT was serially diluted in 10% steer serum for the standard curve, and cow plasma samples were assayed undiluted.

#### Vulva/vagina swab

The vulva was cleared of manure with a paper towel, and the vulva was swabbed using a sterile Dacron swab (#L4363000 Fisher Scientific, Waltham, MA). The “**blind**” vaginal swab was collected in the deep vagina without guided transrectal palpation. The swabs were placed in a 5-ml tube containing 1 ml of 1× PBS and stored at 4°C on blue ice blocks for short-term (3–4 h) storage. Upon return to the laboratory, the tubes were vortexed for 5 s and the swab was removed using sterile forceps. The tubes were stored at −20°C until all samples within a study were collected and then the rbIFNT ELISA was completed.

#### Cervical os fluid collected using an equine swab device (Figure 1)

**Figure 1:**
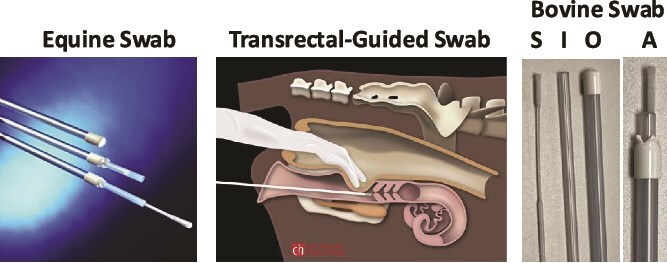
Description of the commercial equine swab (ES) device (left), external os location of cervical sampling (middle), and development of a custom bovine swab (BS) device (right). The custom BS device was constructed with an inner swab (S) that had superior absorbing and releasing features compared to the ES. It was stabilized using a stronger inner tube (I), which allowed entrance into the external os of the cervix and did not bend compared to the equine device. A similar outer protected sheath (O) to the ES was used to prevent the swab from picking up any other fluid when navigating from the vulva to the vagina to the cervix. The fully assembled (A) device was pressed against the cervical external os and opened to collect fluid just inside of the os and prior to the first cervical ring.

External os cervical fluid was collected on days following AI as indicated for each study. The sterile culture swab designed for equine uterine collections (Continental Plastic Corporation, Delavan, WI) was enclosed in a retractable sheath and inserted into the vagina using rectal palpation to avoid the urethra and to direct contact with the external os of the cervix. The sheath was retracted to expose the sterile swab, which was inserted into the cervix approximately one cm. The swab was left inside of the cervix for 10 s to absorb the cervical fluid and then was retracted into the tube. The tube with swab protected inside was then retracted back into the external sheath so that it would close prior to removing the device from the vagina. The tip of the swab was placed in a 5-ml Eppendorf tube with 1 ml of 1X PBS and held at 4°C on blue ice. Upon return to the laboratory, the tubes were vortexed (Eppendorf 5804 R, Hamburg, Germany) for 5 s and the swab was removed using sterile forceps. The tubes were stored at −20°C until all samples had been collected and until the ELISA was performed.

#### Cervical os fluid collected using a custom bovine swab device (Figure 1)

A new swab device was designed for use in cattle, which has an inner tube plastic support (Infusette #B8-5221, Continental Plastics Corporation. Delavan, WI) for the swab so it does not bend when entering the cervix, a highly absorbable Hydra Flock Swab (Puritan, Guilford, ME) and an outer Sani-Shield Protector (#B6-4750, Continental Plastics). By using this smaller swab-head device, it was possible to collect samples just inside of the external os of the cervix.

#### Serum progesterone assay

Serum progesterone concentrations were determined by radioimmunoassay (RIA) as previously described [30]. The LOD of the RIA was 0.02–0.17 ng with intra-assay (7%–15%) and average inter-assay (11%) coefficient of variations.

### Statistics

Interferon-tau concentration in time course experiments of ELISA validation was analyzed using two-way Analysis of Variance (ANOVA) (mixed model). Data were subjected to either repeated measures ANOVA (no missing values) or analyzed by fitting a mixed model as implemented in GraphPad Prism (version 9.0.0 for Windows, GraphPad Software, San Diego, CA, USA, www.graphpad.com). This mixed model used a compound symmetry covariance matrix and is fitted using restricted maximum likelihood. The Geisser–Greenhouse correction was performed by the statistical software used. Šídák’s multiple comparisons test was used to determine the mean differences in pregnancy status on each day tested. Sample sizes are provided in figure legends and tables. Means were considered different if *P* < 0.05. Data are presented as means ± standard error. Absolute IFNT concentrations in the Colorado-BS study (Supplementary Table S1) were measured in the external cervical os and mid-cervical fluids and analyzed using non-parametric Mann–Whitney test. Accuracy parameters (sensitivity, specificity, NPV, PPV, FN, and FP) were compared between external os and mid-cervical samples using the Fisher’s exact test.

## Results and discussion

### Generation of recombinant glycosylated bovine IFNT and polyclonal antibodies

The bIFNT coding cDNA (Supplementary Figure S2) was used to generate rbIFNT in HEK cells that migrated to the expected ~molecular weight (22 kDa) for glycosylated bIFNT [31] on a one-dimensional reducing polyacrylamide gel and cross-reacted with both goat (antibody #51) and rabbit (antibody #5670) anti-rbIFNT polyclonal antibody on western blots (Supplementary Figure S1B). Western blots of rbIFNT were completed to test the reactivity of IFNT polyclonal antibodies in raw serum. The average titers of the primary goat sera were 1:1600 (~47 μg/ml total protein) and rabbit sera were 1:16 000 (4.7 μg/ml total protein).The rbIFNT retained anti-viral activity (2 × 10^8^ IU/mg) using Universal type I IFN (R&D Systems, Minneapolis, MN) as the standard and an in-house antiviral assay [26]. To confirm the identity of the rbIFNT, mass spectroscopy identification of trypsin peptide molecular weights and comparison against the Uniprot_Bovine_101722.fasta file revealed the greatest identity to IFNT2 (also see [25]), Accession A0A3Q1LLH6 (normalized abundance score = 2.7 × 10^11^, 48% coverage, 16 peptide matches, 22.1 kDa, and 6.38 pI) with lesser identity to IFNT3 and IFNTc1.

### IFNT enzyme-linked immunosorbent assay

Conditions were optimized to develop a 5 h IFNT ELISA with a range of detection between 40 and 600 pg/ml and an LOD that ranged from 30 to 70 pg/ml depending on the assay and the plate examined. The LOD for these assays averaged 54 pg (2× the OD450 of steer serum control), which was determined for each plate. For the 100 pg/ml rIFNT quality control standard, inter-assay coefficient of variation was typically 9.2%–12.5% and intra-assay coefficient of variation was 0%–7.2%. These coefficients of variation were calculated for each plate and each experiment.

**Figure 2:**
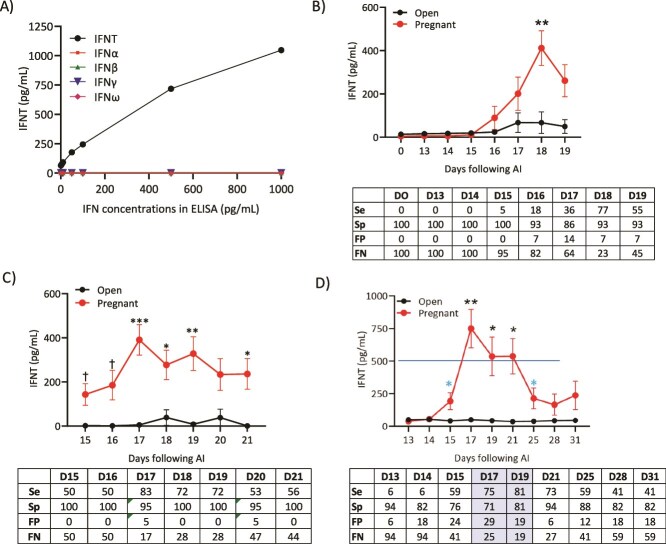
Determination of IFNT antigen specificity, IFN profiles, and accuracy estimates when detecting pregnancy status based on IFNT concentrations (open and pregnant cows) on days indicated compared to Day 32+ US. (A) IFNT ELISA antigen specificity. The bovine IFNT ELISA detected only IFNT and no other related type I IFNs or the more distant type II IFNG. (B) Detection of IFNT in blind vaginal swabs (*n* = 28 cows). There was 100% accuracy in specificity and FN calls on day of AI (Day 0). The OCT is based on the lack of detection of IFNT on days that IFNT is not expected to be present (Days 0–14). It is apparent from this study that IFNT is not present in blind vaginal swabs until about Day 16 following AI. There were no FP cows on Days 0–15 for this reason (Day *P* < 0.0001, Pregnancy status *P* < 0.0058, Day × Pregnancy status *P* < 0.0001). (C) IFNT concentrations in transrectal guided cervical external os samples and accuracy data using the equine swab (ES) device on Days 15–21 following AI (*n* = 37 cows) (Day *P* < 0.05, Pregnancy Status *P* < 0.001, Day × Pregnancy Status *P* > 0.05). (D) IFNT concentrations in transrectal guided external cervical os ESs in 34 lactating dairy cows on Days 13 to 31 following AI. This was a qualitative IFNT analysis. The line represents the highest concentration (500 pg/ml) in the linear range of the assay. Values above this line are not precise estimates of IFN concentrations. For accuracy estimates expanded on Day 17, see Table 1. Values represent average IFNT concentrations ± SE. The blue “*” represents a *t*-test at *P* < 0.05 when protected by the significant Day × Pregnancy Status (Day *P* < 0.0002, Pregnancy Status *P* < 0.0001, Day × Pregnancy Status *P* < 0.0001) (^*^P < 0.05, ^*^^*^P < 0.01, ^*^^*^^*^P < 0.001, †*P* < 0.10). Note that the actual absorbance value from the ELISA was used in these studies. There were also a few FP cows (detected IFNT concentrations greater than LOD on days tested but not pregnant (open in the graphs) at US. This is why there may appear to be a slight increase in IFNT concentrations after Day 17 in the cows determined to be open by US in Panels B and C. This was done in this figure to illustrate how FP cows can influence IFNT levels prior to US. For this reason, in all subsequent analyses, any cows with IFNT levels at or below the LOD on days tested were designated as 0 pg IFNT/ml.

Other type 1 IFN proteins such as IFNα, IFNβ, IFNω, and IFNγ were tested on the same 96-well plate with IFN concentrations of 1000, 500, 100, 50, 10, 5, and 1 pg/ml (Figure 2A). The IFNT ELISA using the anti-bIFNT antibodies detected only rbIFNT and did not cross-react with the other type 1 or type II IFN family members at any of the concentrations tested.

#### Blood and milk samples

Interferon-tau was not detected in blood, milk, or vulvar fluid samples using the IFNT ELISA on Days 5–21 following AI in dairy cows retrospectively confirmed to be pregnant using US on Day 32, even with an LOD of 30–50 pg/ml (data not shown).

#### Vulvar and vaginal swab studies using a simple cotton swab device

Vulvar, vaginal, and cervical fluids were examined. IFNT has been reported to be produced in levels as high as 100 μg/24 h from Day 15 to 17 ovine conceptuses cultured in vitro [32]. Averages of 263 (*n* = 8, [22]) to 765 (*n* = 6, [33]) ng/ml of uterine flushing from Day 16 pregnant cows are documented (also see: [34]). Because a cervical plug does not form in the bovine cervix following fertilization, it was anticipated that IFNT might leak from the uterus through the cervix into cervical, vaginal, and vulva fluids, which could be collected with an absorbent swab. Collection of fluid from the cervix, vagina, and vulva using a swab was also considered advantageous compared to the collection of blood samples that require needles, vacutainers, and a centrifuge.

A pilot study (*n* = 20 cows) was completed on Day 18 following AI using a blind (i.e., not directed with transrectal palpation) deep vaginal swab. The vulva was cleaned with a paper towel, and vaginal secretions were collected with a simple polyester-tipped ES (Continental Plastics Corp., Delavan, WI; Figure 1). Interferon-tau was detected by the ELISA in blind deep vaginal swab samples from some cows diagnosed as pregnant by US on Day 32 with a 13% FN rate (not shown). In an expanded time course, blind deep vaginal samples were collected from Days 0 to 19. Interferon-tau was successfully detected in blind deep vaginal samples (Figure 2B). There was a 0% FP rate and a 100% FN rate, and no FP samples were collected on Days 0–15 prior to AI. These results and timing are supported by studies that show that IFNT is not produced in significant amounts until the trophoblast forms [18] and begins expansion/elongation around Days 13–14 [35]. The lack of detection of IFNT by Day 15 in cervical/vulvar/vaginal samples may also reflect the inability to measure IFNT because of lower concentrations at the site of sampling in the deep vagina that is further from the source (release from the conceptus in the uterus and leakage from the cervix into the vagina). There was a 100% specificity in identifying cows with no conceptus, but a 0% sensitivity in identifying pregnant cows prior to Day 15 following AI when compared to US results on Day 32. For samples collected after Day 15, FN rates improved and ranged from 23% to 82% on Days 16 to 19 following AI with the lowest rate at 23% on Day 18. These FN rates are unacceptable, especially when considering resynchronization using PGF. Consequently, it was reasoned that samples from the external os of the cervix might have greater IFNT concentrations and be more accurate because they represent closer proximity to the source of IFNT from the conceptus in the uterus.

#### Collection of external cervical os fluid samples using a commercial equine swab device

To improve the accuracy when identifying non-pregnant cows with blind vaginal swab samples, transrectally-guided swab samples of the external cervical os fluid were collected using a commercial ES device. As shown in Figure 2C, external os swab samples were collected from Days 15 to 21 (*n* = 37 cows) and tested using OCT. The IFNT was detected in swab samples as early as Day 15 and as late as Day 21 in this study. The greatest concentration of IFNT appeared to be on Days 17–21. The FN rate on Day 17 was 17% and on Days 18–19 was 28%. Because there was significant detection of IFNT on Day 15, a more expanded time course following AI was completed from Days 13 to 31 (*n* = 34 cows; Figure 2D). In this study, IFNT was consistently detected on Days 15–25 in pregnant compared to open cows. The greatest amount of IFNT in external cervical os fluid appeared to be on Day 17 with a 25% FN rate in this study (see Table 1 for other accuracy estimates associated with Figure 2D).

**Table 1:** Calculated parameters for open cow test (OCT) results based on pregnancy diagnosis by ultrasound in dairy cows after insemination (CO, USA).

Endpoints	Definition	Cows (ES, AI) (OCT Day 17) (*n* = 34)	Cows (ES, AI) (OCT Day 17) (*n* = 323)	Cows (BS, AI) (OCT Day 17) (*n* = 99)
		Value	Value	Value
TP (*n*)	P by OCT and US	12	92	35
TN (*n*)	NP by OCT and US	12	168	18
FP (*n*)	P by OCT and NP by US	5	38	44
FP (%)	(FP/FP + TN)	29.4	18.5	71
FN (*n*)	NP by OCT and P by US	5	25	2
**FN (%)**	**(FN/FN + TP)**	**29.4**	**21.4**	**5.5**
ACC (%)	(TP + TN/*n*)	70.5	80.5	53
Se (%)	(TP/TP + FN)	70.6	78.6	94.5
Sp (%)	(TN/TN + FP)	70.6	81.5	29
PPV (%)	(TP/TP + FP)	70.6	70.7	44.3
**NPV (%)**	**(TN/TN + FN)**	**70.6**	**87**	**90**
EEM (%)	(FP/TP + FP + FN)	22.7	24.5	54

P, pregnant; NP, non-pregnant; TP, true positive; TN, true negative; FP, false positive; FN, false negative; ACC, accuracy; Sp, specificity; Se, sensitivity; PPV, positive predictive value; NPV, negative predictive value; EEM, estimated embryonic mortality; AI, artificial insemination; ES, equine swab device; BS, bovine swab device; US, ultrasound. The bold text and numbers represent the most important accuracy estimates for the open cow test.

Day 17 was identified as the best day to collect samples for a larger clinical (*n* = 323 cows, Dairy B) trial and provides the opportunity to resynchronize estrus prior to the next estrous cycle (~Day 21). In this clinical trial, there was a 35% pregnancy rate based on US on Day 32. The FN rate was 21.4% with an NPV of 87% (Table 1).

### Development of a custom bovine cervical swab device and testing dairy cows for accuracy using open cow test

**Figure 3:**
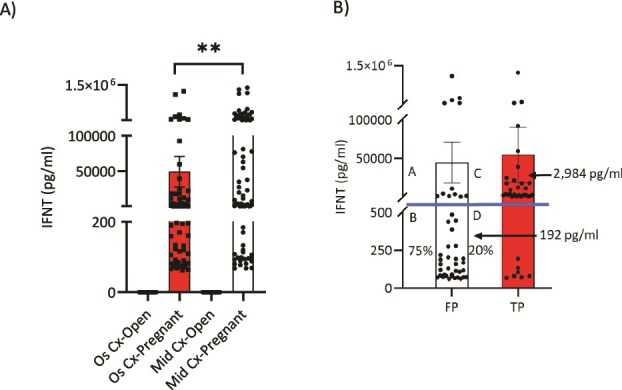
IFNT concentrations using the custom BS device in external cervical os (Os Cx) compared to mid-cervix (Mid Cx, passed the first ring) on Day 17 open and pregnant cows (*n* = 99) (A) and distribution of IFNT concentrations and median values in FP compared to TP cows based on US on Day 32 (B). Swab samples were collected from the same cows. The external swab sample is inside of the os but prior to the first ring. The mid-cervical swab sample is a sample after the first cervical ring. The external swab sample was collected first and then the mid-cervical swab sample was collected using two separate BS devices. IFNT concentrations were 49,210 ± 21 261 pg/ml for Os Cx-Pregnant cows and 151,299 ± 34 479 pg/ml for Mid Cx-Pregnant cows. (B) Distribution of IFNT concentrations in external cervical os swab samples (Os Cx) on Day 17 in FP (FP/embryo mortality) and TP dairy cows called by US (BS; Table 1). Notice the high and low clusters of IFNT concentrations in both groups but also the much lower median concentration with 75% of values in the lower quadrant in FP pregnancies. Median concentrations of IFNT are shown by arrows for FP and TP cows (^*^^*^P < 0.01). The line shows the separation between high and low clusters. The LOD for this assay was 64 pg/ml. Data are quantitative. Values represent the mean ± SE.

To reduce the 21.4% FN rate in the clinical trial in Table 1 (Colorado, *n* = 323), changes in the method of collection and the composition of the swab device were made (Figure 1). A new BS device was developed with superior absorptive properties and tested by collecting external os and mid-cervical samples on Day 17 in 99 cows (Figure 3A). The IFNT concentrations data are quantitative in this study, as shown in Table 1 (99-cow study). For example, samples with IFNT concentrations ≥600 pg/ml were diluted until IFNT concentrations were within the linear range (40–600 pg/ml) of the assay and then the values were multiplied by the dilution factor to quantitate IFNT concentrations. Internal cervical (i.e., mid-cervical swab sample) concentrations of IFNT in pregnant cows were about 3× greater (*P* < 0.01): IFNT concentrations were 49 210 ± 21 261 pg/ml for external cervical os samples and 151 299 ± 34 479 pg/ml for mid-cervical samples from the same pregnant cows (Figure 3A). There was no difference in accuracy estimates between inner and outer cervical swab determinations of IFNT concentrations (Supplementary Table S1). For example, the FN rate was 5.4% for swabs collected from the internal and external cervical os samples. Collection of cervical fluid with the swab device regardless of location had no influence on pregnancy rate compared to other contemporary cows inseminated at the dairy either prior to or after this study (data not shown). Consequently, all subsequent studies were performed using external cervical os samples for ease of collection by the technician and comfort of the cow.

The accuracy of the OCT test in correctly detecting open cows increased with these changes in the method of swab collection, composition of the swab, and the ELISA assay (Table 1). The FN values (Table 1) started at 29.4% in the 34-cow time-course study and decreased to 21.4% in the 323-cow clinical study. With the adoption of the new custom BS device and collection of the external cervical os secretions, the FN rate improved to 5.5% in the 99-dairy cow study.

**Table 2:** Calculated parameters for open cow test (OCT) results based on pregnancy diagnosis by ultrasound in beef heifers and cows after insemination or embryo transfer in five different studies (USDA-ARS, MT, USA).

Endpoints	Definition	Heifers (ES, AI) (OCT Days 18–20) (*n* = 102)	Cows (ES, AI) (OCT Day 18) (*n* = 135)	Cows (ES, ET) (OCT Day 18) (*n* = 126)	Cows (BS, AI) (OCT Day16) (*n* = 70)	Cows (BS, AI) (OCT Day 18) (*n* = 66)
		Value	Value	Value	Value	Value
TP (*n*)	P by OCT and US	23	75	33	28	23
TN (*n*)	NP by OCT and US	66	47	80	36	39
FP (*n*)	P by OCT and NP by US	3	5	11	5	4
FP (%)	(FP/FP + TN)	4.4	9.7	12.1	12	9.3
FN (*n*)	NP by OCT and P by US	10	8	2	1	0
**FN (%)**	**(FN/FN + TP)**	**30.3**	**9.7**	**5.7**	**3.4**	**0**
ACC (%)	(TP + TN/*n*)	87.3	90.3	89.6	91.4	94
Se (%)	(TP/TP + FN)	69.6	90.3	94.3	96.6	100
Sp (%)	(TN/TN + FP)	95.6	90.3	87.9	87.8	90.7
PPV (%)	(TP/TP + FP)	88.4	93.7	75	84.8	85.2
**NPV (%)**	**(TN/TN + FN)**	**86.8**	**85.4**	**97.5**	**97.3**	**100**
EEM (%)	(FP/TP + FP + FN)	8	6	24.4	14.7	14.8

P, pregnant; NP, non-pregnant; TP, true positive; TN, true negative; FP, false positive; FN, false negative; ACC, accuracy; Sp, specificity; Se, sensitivity; PPV, positive predictive value; NPV, negative predictive value; EEM, estimated embryonic mortality; AI, artificial insemination; ES, equine swab device; BS, bovine swab device; US, ultrasound. The bold text and numbers represent the most important accuracy estimates for the open cow test.

### Identification of non-pregnant beef heifers and cows based on the lack of detection of IFNT in external cervical os samples using the commercial equine swab device (Table 2)

False negative rates were determined for external os cervical swab samples collected on Day 19 post-estrus using beef heifers (*n* = 6) that were not artificially inseminated to serve as negative (open) controls using the commercial ES device. The assay was 100% (6/6) accurate (0% FN) when classifying these heifers as open. As in dairy cows (*n* = 28; Figure 2B), IFNT was not detected in samples collected on Day 0 (day of AI) from beef heifers with a 0% FN rate and no FP samples as expected because IFNT is not produced until the trophoblast forms [18] and begins expansion/elongation around Days 13–14 [35]. The FN rate was 30% (10/33, Table 2) for swab samples collected on Days 18, 19, or 20 from heifers determined to be pregnant by US on Day 32 (*n* = 102). The accuracy of the OCT in identifying non-pregnant beef heifers (specificity) on Days 18–20 was 96%. The 30% FN rate in heifers is not acceptable and is likely due to the anatomical nature of the cervices in heifers, which have not yet experienced the dilation needed to deliver a calf and, therefore, are less likely to allow uterine fluid to enter the cervix.

Swab samples from beef cows collected on Day 18 (*n* = 135) post-AI had an FN rate of 9.7% (8/83) with a 90% accuracy in identifying non-pregnant cows (Table 2). Samples from beef cows (*n* = 126) that received a Day 7 frozen/thawed embryo following embryo transfer on Day 7 with cervical fluid samples collected on Day 18 had an 87.9% specificity and an FN rate of 5.7% (2/35, Table 2). All cows in this study were technically pregnant on Day 7, the day of embryo transfer. This is reflected by the specificity of 87.9% and the NPV of 97.5%.

### Identification of non-pregnant beef cows based on the lack of detection of IFNT in external os samples using the custom bovine swab device

Beef cows were sampled using the new BS device on Days 16 (*n* = 70) and 18 (*n* = 66) (Table 2), which resulted in a 96.6% sensitivity (3.4% FN rate) on Day 16 and 100% sensitivity (0% FN rate) on Day 18. These results are interpreted to reflect better absorption of cervical fluid by the customized BS and, therefore, improved accuracy for the detection of IFNT compared to the commercial equine device.

### Detection of pregnancy and embryonic mortality through external cervical os IFNT and serum progesterone concentrations

**Figure 4:**
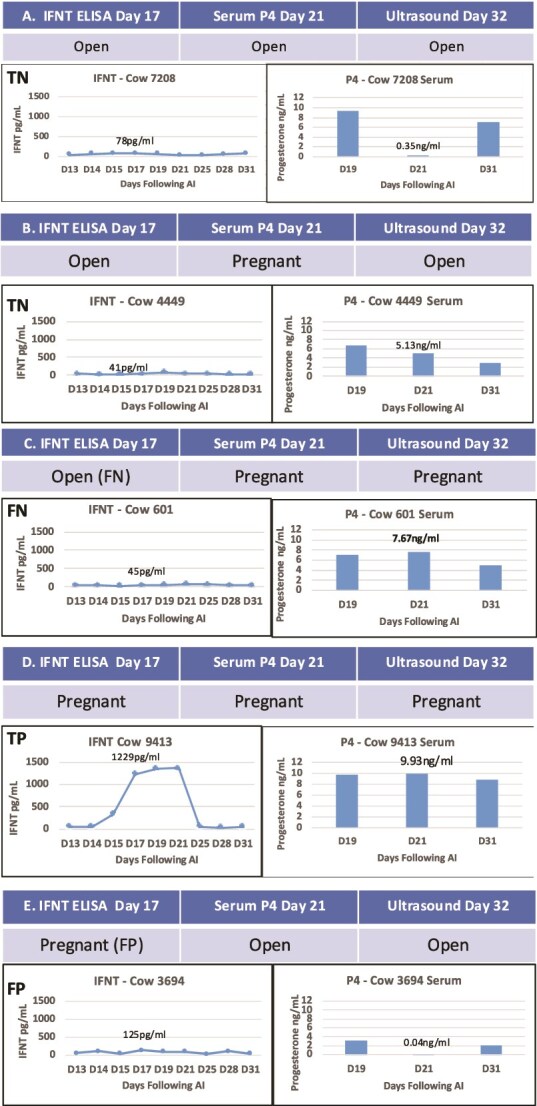
Concentrations of IFNT and progesterone in TN (A and B), FN (C), TP (D), and FP (E) cows. The FP cows experienced embryonic mortality any time between OCT with a detectable IFNT concentration on Day 17 and US diagnosis on Day ≥32.

Identification of non-pregnant cows or cows with embryonic mortality can be based on the presence of IFNT on Day 17 and either serum progesterone concentrations that are higher (later mortality) or lower (early mortality) than 1 ng/ml on Day 21 following AI (Figure 4). The IFNT data in Figure 2D were analyzed to profile true and false positive and negative dairy cows (Figure 4). The IFNT data from two groups of cows determined to be open by US on Day 32 (true open) were analyzed. The first group of true open cows (Figure 4A: 10/34 cows) had Day 17 IFNT concentrations below the LOD, Day 21 serum progesterone less than 1 ng/ml, and an embryo was not detected by US on Day 32. The serum progesterone concentrations on Day 21 in these cows were within the range of 0.04–1.1 ng/ml. In the second set of true open cows (Figure 4B: 7/34 cows), IFNT concentrations were below the LOD of the ELISA, progesterone concentrations on Day 21 were >1 ng/ml, and US revealed that these cows were not pregnant. These TN cows probably had extended cycles and, therefore, a functional CL at the time of blood collection on Day 21. Several FN cows were diagnosed as open by OCT on Day 17 but had high progesterone on Day 21 and were found to be pregnant by US (Figure 4C: 6/34 cows). These FN cows pose a problem for resynchronization using PGF because lysing the CL based on the OCT diagnosis would abort the pregnancy.

Many cows were called TP based on high IFNT levels on Day 17, high serum progesterone (>1 ng/ml) on Day 21, and the presence of an embryo with a heartbeat at US on Day 32 (Figure 4D: 11/34). The final group represented in Figure 4E are the FP cows that had detectable IFNT, with low serum progesterone on Day 21 and lack of an embryo at US on Day 32. Because the ELISA is very sensitive for IFNT, cows with any amount of IFNT over the LOD would be called pregnant. However, some of these cows were not diagnosed pregnant by US with the possible interpretation that these cows were experiencing embryonic mortality at the time of OCT. In other FP cases (not observed in this study), cows may have detectable IFNT on Day 17 followed by lysis of the CL between Day 17 and the time of US.

The addition of concurrent progesterone and PSPB/PAG1 measurements in future studies may help better understand the ratio of embryonic mortality among FP cows. Please see Supplementary Figure S3 for comparison of PSPB compared to IFNT concentrations in swab samples depicted in Figure 2D. It was apparent from this preliminary study that in cervical swab samples, PSPB was not accurately detected on Days 13–28 in pregnant cows (e.g., no differences in PSPB between open and pregnant cows) when using external os swab samples (equine device) until an appreciable increase was observed on Day 31, although the detection of PSPB in blood may be accurate as early as Days 20–23 when considering more than one sampling day [36]. Herein, there were many pregnancies that were detected based on the presence of conceptus-derived IFNT but were lost by the day of US. For example, 11 beef cows that received embryos on Day 7 (i.e., pregnant) and had high IFNT concentrations on Day 18 were later found to be open by US.

### Study of pregnancy by using violin distribution plots

**Figure 5:**
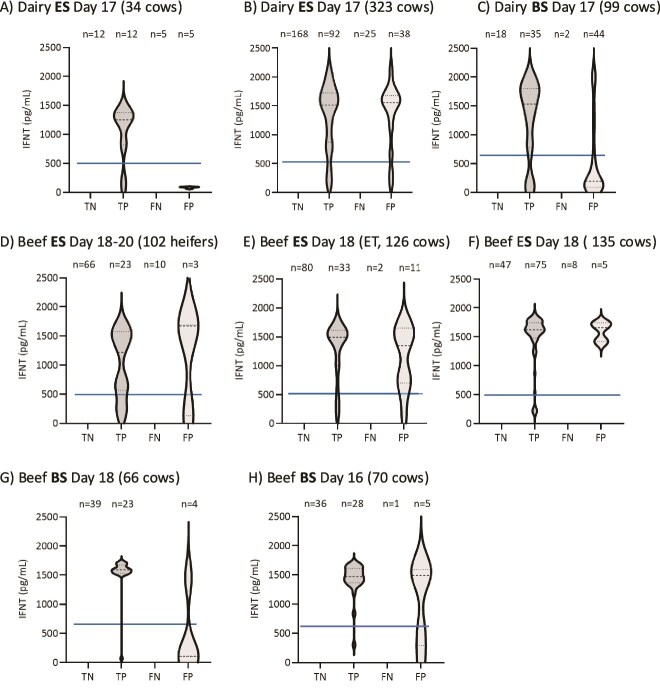
IFNT “Violin” profiles on Day 17 in TN, TP, FN, and FP cattle in the studies described in Tables 1 and 2. Columns represent the analysis of IFNT on Day 17 using either the equine swab device or the new BS. Notice that most of the FP dairy cows had very high IFNT levels indicative of presence of a conceptus at the time of sample but then lost the pregnancy (embryo mortality) by the time of US at 32 days. The line represents the highest concentration in the linear range of the assay. Values above this line are not precise estimates of IFN concentrations.

The distribution of IFNT concentrations in open compared to pregnant cows using the equine and BS devices is provided in Figure 5 for both dairy (Table 1) and beef (Table 2) cows tested. The violin plots show the distribution of IFNT concentrations in the TN, TP, FN, and FP groups of cattle identified. As expected, IFNT was not detected in TN or FN cows. In most all FP cattle, there is a very high IFNT concentration cluster in the upper quadrant of this group, indicating the presence of a conceptus on the day of OCT. However, the embryo was no longer present at US on Day 32 because of embryonic mortality. Likewise, there was a smaller population of females with a low cluster of IFNT concentrations in the FP group. This “bimodal” distribution of IFNT concentrations—a low cluster and a high cluster—was also evident in the TP cows. The basis for these two clusters of IFNT concentrations while factoring in serum progesterone and PSPB concentrations with blood cell and cervical fluid proteomics may illuminate the timing and reasons for embryo mortality in future studies. It is evident that values greater than the LOD represent the detection of IFNT from a conceptus, and this is not a problem with the background of the assay. In most all TP females, there were clearly two clusters of IFNT concentrations, with 75% of cows having high concentrations, while 20% of cows had values closer to the LOD, but all cows maintained their pregnancies (Figure 3B). In the FP group, almost all cows have similar IFNT profiles compared to TP cows. Because IFNT is specific to pregnancy, these FP cows identified by OCT to be pregnant on Day 17 experience embryonic mortality, which cannot currently be avoided. The rate of embryonic mortality fluctuates between 40% and 60% with different farms and management strategies [1, 27]. It follows that FP cows in these independent studies experienced embryonic mortality, which decreased the sensitivity of the OCT.

### Use of the IFNT enzyme-linked immunosorbent assay to study embryonic mortality (false positive cows)

#### Dairy cows

Overall FP IFNT did not differ from TP IFNT in the study where the BS was used to collect external os cervical fluid in dairy cows (*n* = 99; Table 1 and Figure 3B). This was because there was such a massive range of IFNT values in both groups. However, about 75% of the FP IFNT values were below 500 pg/ml, whereas only 20% of the TP cows had values below 500 pg/ml (Figure 3B). Thus, there are clearly two populations of FP and TP based on IFNT concentrations. For interpretation of each of these IFNT clusters, see Table 3. The majority of FP (predicted to have embryonic mortality) cows identified in Table 1 have variable concentrations of IFNT with a median level that is much lower than TP cows (Figure 5A–C). These natural variations in IFNT concentrations may provide insights into which cows are experiencing embryonic mortality and why these embryos are dying. Embryonic loss might be predicted based on IFNT concentrations in cervical os fluid when using the new and improved BS device as demonstrated in the dairy cow study (*n* = 99; Table 1 and Figures 3B and5C). Median IFNT concentrations were 192 pg/ml in FP cows (*n* = 44) compared to 2984 pg/ml in TP cows (*n* = 35; Figure 3B) on Day 17. By definition, 75% of the FP cows (33/44 cows) had IFNT concentrations that were below the median concentration of 192 pg/ml (Figure 3B). However, only 20% of the TP cows (7/35 cows) had IFNT concentrations lower than the median concentration (192 pg/ml) of the FP cows. Notice the high and low clusters of IFNT concentrations in both groups but also the much lower median concentration with 75% of values in the lower quadrant in FP pregnancies. Through examining days earlier and later than Day 17 in future studies using the BS device, these differences in IFNT concentrations may be more pronounced when comparing FP to TP cows and will provide greater insights into timing and mechanisms of embryonic mortality compared to analysis of Day 17 following AI alone (Figure 3B).

**Table 3:** Comparison of IFNT concentrations in false positive (FP) cows with high (A) and low (B) IFNT concentrations compared to true positive (TP) cows with high (C) and low (D) IFNT concentrations^a^ (99 cow study in Table 1).

Case		
A	FP/high IFNT (11/44, 25%)	High IFNT concentrations were detected on Day 17; however, these FP cows did not maintain pregnancy. The reasons could be that early developing conceptuses secreted high amounts of IFNT on Day 17 but did not release enough IFNT to block the endometrial luteolytic response prior to Day 17. Or the CL formed was inferior and could not maintain the production of progesterone
B	FP/low IFNT (33/44, 75%)	Low concentrations of IFNT may not have been able to signal maternal recognition of pregnancy by Day 17. Conceptuses may not have been developed enough to secrete enough IFNT to prevent luteolysis by Day 17. The CL also may not have been robust enough to maintain an endometrial environment conducive to maintain pregnancy
C	TP/high IFNT (28/35, 80%)	The concentrations of IFNT and progesterone were high enough to maintain pregnancy in these TP cows
D	TP/low IFNT (7/35, 20%)	Concentrations of IFNT low, but high enough before, during, and after Day 17 to maintain pregnancy

^a^The scenarios of A, B, and D require investigation through the time of maternal recognition of pregnancy to determine the causes of embryonic mortality by measuring IFNT and progesterone daily. The study of both IFNT and plasma progesterone concentrations from Days 15 to 21 may help resolve some of these relationships and solve the puzzle in context of the importance of conceptus-derived IFNT as well as CL-derived progesterone when maintaining early pregnancy in cattle.

#### Beef cattle

Similar to dairy cows, there tended to be two populations of IFNT clusters in TP and FP beef heifers and cows (Figure 5D–F) when using the commercial ES device. Notably, when using the new bovine device on Days 18 (Figure 5G) and 16 (Figure 5H) following AI, the clustering was still apparent.

### Receiver operator curve analysis of pregnancy diagnosis using the OCT

The receiver operator curve (ROC) analysis [37] (Supplemental Figure S4) is focused on how well a diagnostic performs to identify the affected group vs normal (i.e., infected vs. control; or pregnant vs. non-pregnant/open). The ROC analysis in cattle using the bovine compared to the ES device seemed to better predict specificity compared to sensitivity (Supplementary Figure S4). This is likely caused by a much lower FN rate when using the BS compared to the ES device. It was clear that the performance of the assay in context of area under the ROCs improved over time and when changing from equine to BS devices in dairy cows (Supplementary Figure S4A–C). In the most recent beef cow studies (Supplementary Figure S4G and H), area under the ROC was 0.996 on Day 18 and 0.93 on Day 16 following AI when swabbed with the BS device and improved compared to beef cattle swabbed with the ES device (Supplementary Figure S4D–F).

It is very clear that this analysis may vary from farm to farm. Those operations with greater pregnancy rates may not benefit as much as those with lower pregnancy rates. In addition, there may be differences in embryonic mortality rates from farm to farm. It is evident that increased embryonic mortality will impact the sensitivity of the assay, but this may be an advantage of the assay if we can predict embryonic mortality. In the future, it will be possible to study cervical IFNT and blood progesterone as well as PSPB concentrations from Days 15 to 32, which will not only provide information on when conceptuses are producing enough IFNT to be detected in external cervical os samples but also how long the IFNT, progesterone, and PSPB/PAG1 concentrations stay elevated and when they decline to determine the timing of early (Days 15–21) and late (>Day 21) embryonic mortality.

### Comparison of IFNT with Interferon-Stimulated Genes (ISGs) as markers for pregnancy

The use of IFNT to identify open cows rather than ISGs is indicated when detecting presence of a conceptus because IFNT is specific to the trophectoderm cells of the conceptus, whereas ISGs such as ISG15 and ISGylation are stimulated in the endometrium by IFNT and are also induced by many other immune and inflammatory signals [38–40]. Regardless, previous studies using white blood cell ISG mRNAs [41–43] and a more recent study using cytobrush cervical samples yielding RNA [27] revealed that by using RT-qPCR ISGs such as ISG15 provided a useful marker for pregnancy status. It was clear in the earlier studies that ISG mRNA concentrations in white blood cells were elevated during early pregnancy in ruminants. However, there was consistent variability in ISG15 that was mostly not explained and consequently contributed to a high FP rate (for review see: [7, 44]). More direct analysis of ISGs in cervical cell RNA revealed improvements in accuracy, and when analyzed in addition to serum progesterone and PAG1 profiles, this provided a reasonable approach to study embryonic mortality [27]. ISG concentrations increase in the female reproductive tract when sampled from the vulva to the cervix [27], which was similar to the increase in IFNT concentrations from the vulva compared to the cervix in the present study. The reported FN rate of 4.4% in the 86-cow study by Dominques et al. [27] was impressive, especially in context of using ISGs, which are known to fluctuate within and across dairies. Furthermore, in a recent collaborative study by Newman et al. (from the Bisinotto laboratory at University of Florida, manuscript in preparation), external cervical os concentration of IFNT was correlated with ISG15 transcript abundance in cervical cytology of pregnant cows, but not with ISG15 transcript abundance in cervical cytology of non-pregnant cows or in Peripheral Blood Mononuclear Cells (PBMC) (except for *Mx2* in pregnant cows). External cervical os IFNT concentrations were greater than the vaginal fornix concentration of pregnant cows, confirming that the external cervical os is the most appropriate site for the collection of swab samples for early pregnancy diagnosis. In addition, it was confirmed in this study that collecting external cervical swabs did not impair pregnancy rates. It is of interest to determine consistency in the FN rates in future cervical external os ISG and IFNT studies when using the new BS device.

### Preliminary market analysis of open cow test to identify open cows so that they can be resynchronized and inseminated by Day 21

A current market analysis is needed using today’s costs to accurately predict the net worth of the OCT in the context of resynchronization of estrus and second AI of open cows by Day 21. The key factors for this analysis are pregnancy rate, NPV, current costs to maintain open cows, and the cost of completing the diagnostic (currently estimated at $3 per cow). With utilization of the new BS device, it is now possible to achieve 0%–5% FN rates, which reduces the economic loss when aborting pregnant cows following resynchronization using PGF. The average accuracy values across all studies using the new BS device herein (235 cows total: 99 dairy and 136 beef cows) were estimated to be 96.6% sensitivity, 3.4% FN, 96.8% NPV, and 63.7% specificity (Tables 1 and 2). Cabrerra [45, 46] estimated costs to maintain open cows at $47 per week in 2012. These costs have escalated because of inflation and energy costs associated with cattle production systems. For example, based on the conservative inflationary calculator at the U.S. Bureau of Labor Statistics, costs may be greater than $65 per week to maintain open cows presently.

### Concluding remarks (see graphical abstract)

After interviewing about 30 dairy veterinary medicine practitioners, professors, and animal biopharma professionals in a Research to Market short course hosted by Colorado State University STRATA, it was clear that everyone was very concerned about reducing embryonic mortality and more rapid (earlier in the lactation cycle) management of re-breeding the early non-pregnant (open) cow. The primary take-home lesson was that most everyone was initially focused on identifying the pregnant cows and the high FP rate on Day 17 because of embryonic mortality. It was argued effectively that these cattle must be checked again at Day 32+ to confirm pregnancy status. While the focus on the early pregnant cow is of interest in the context of finding cows undergoing embryonic mortality, the utility of identifying the open cows was not completely appreciated. Upon explaining the importance of managing the open cows identified by Day 17 following the first AI through the resynchronization of open cows so that they can be re-inseminated by Day 21 (depending on protocol applied) compared to industry standards at Day ~39, most all agreed that if the FN rates were low (i.e., 5%) at Day 17 OCT, then resynchronizing open cows by Day 21 would certainly be more feasible.

In the current application following a Double Ovsynch for the first AI following calving, it would be feasible to test cows on Day 17 using OCT, with PGF given for open cows on Day 18 (PM), GnRH on Day 20 (PM), and AI on Day 21 (AM) in future studies. An alternative approach for resynchronization is injection of PGF on Days 19 and 20 in the AM and 32 h after the second PGF, injecting GnRH (i.e., Day 21 in the afternoon) with AI in AM on Day 22 (16 h later). This protocol was used in a resynch program after US confirmed a corpus luteum (CL) and open pregnancy diagnosis on Day 25 [47]. As the Day 19 cows are tightly synchronized prior to the first AI, in this scenario, there may be a need for only one treatment of the PGF with the OCT.

The time course study of cervical os IFNT concentrations in Figure 2D using the ES device needs to be repeated with the more sensitive BS device because the bovine device collects more IFNT and is more accurate in the context of lower FN values (see Tables 1 and 2). Larger clinical trials are needed in dairy and beef cattle to better estimate and validate the consequences of loss of pregnancy in any pregnant cows that might be aborted by using PGF when using the BS device. Furthermore, the net worth of an OCT implemented on Day 16 or 17 following the first AI needs to be determined using current dairy and beef cow economic indicators. Likewise, organic dairies may be interested in using OCT to manage open cows because they are hormone-free operations.

Finally, the ability to accurately track embryonic mortality using IFNT concentrations and the sorting of these pregnancies into low and high IFNT clusters is novel. These IFNT data in addition to profiling progesterone and PSPB concentrations as well as potentially the white blood cell proteome and transcriptome may bring clarity in future studies as to when and why embryonic mortality occurs. Also, the discovery of future biomarkers for cattle with embryonic mortality and/or impaired embryonic competence may be used to better manage and improve reproductive management.

## Supplementary Material

Supplemental_Figure_1_ioaf066

Supplemental_Figure_2_ioaf066

Supplemental_Figure_3_ioaf066

Supplemental_Figure_4_ioaf066

Supplemental_Table_1_ioaf066

Supplementary_Figure_Legends_ioaf066

## Data Availability

Data are publicly available and are stored on backup drives at Colorado State University, Department of Biomedical Sciences, Animal Reproduction and Biotechnology Laboratory under the OCT Data identifier.
