# Correction to: Early identification of bovine pregnancy status and embryonic mortality

**DOI:** 10.1093/biolre/ioag105

**Published:** 2026-05-19

This is a correction to: Jeanette V Bishop, Aydin Guzeloglu, Tom Scheller, Joshua J Docheff, Carolina L Gonzalez-Berrios, Hana Van Campen, Terry M Nett, Abigail L Zezeski, Thomas W Geary, William W Thatcher, Thomas R Hansen, Early identification of bovine pregnancy status and embryonic mortality, *Biology of Reproduction*, Volume 112, Issue 5, May 2025, Pages 981–995, https://doi.org/10.1093/biolre/ioaf066

There are two relevant patents describing use of interferon-tau diagnostics as a method of pregnancy determination, which the authors failed to disclose as conflicts of interest in the original manuscript submission.

The first patent is held by the corresponding author and the seventh co-author: Early determination of pregnancy status in ruminants, WO EP US US10288625B2, 2019

The second patent is held by the corresponding author, and the first, second, and sixth co-authors: Open cow test, WO WO2024107797A1, 2024

The Conflict of interest statement is amended to: “The corresponding author holds the patents Early determination of pregnancy status in ruminants, WO EP US US10288625B2, 2019 and Open cow test, WO WO2024107797A1, 2024. The seventh co-author also holds patent WO EP US US10288625B2, 2019 and the first, second, and sixth co-authors also hold patent Open cow test, WO WO2024107797A1, 2024”.

The Editors have since reassessed the paper considering these late disclosures, and issued this Correction Notice.

The authors apologize for these oversights.

