# Supplementary figures and images for: Early identification of bovine pregnancy status and embryonic mortality
Source: Biol Reprod. 2025 Mar 28;112(5):981–95. doi: 10.1093/biolre/ioaf066 (PMC12078079; doi:10.1093/biolre/ioaf066)

A IFNT (bTP1 509) cDNA

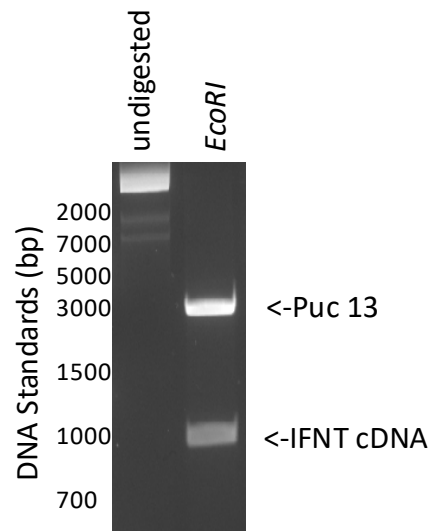

B. Western Blot

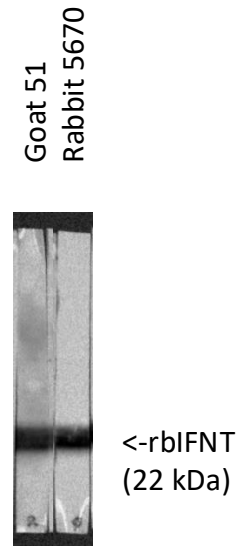

Supplement: Supplemental_Figure_1_ioaf066 [file supplemental_figure_1_ioaf066.pdf]

**Plasmid Map**

pD2529-CMV:220531

Only single and double cutters are shown in the map.

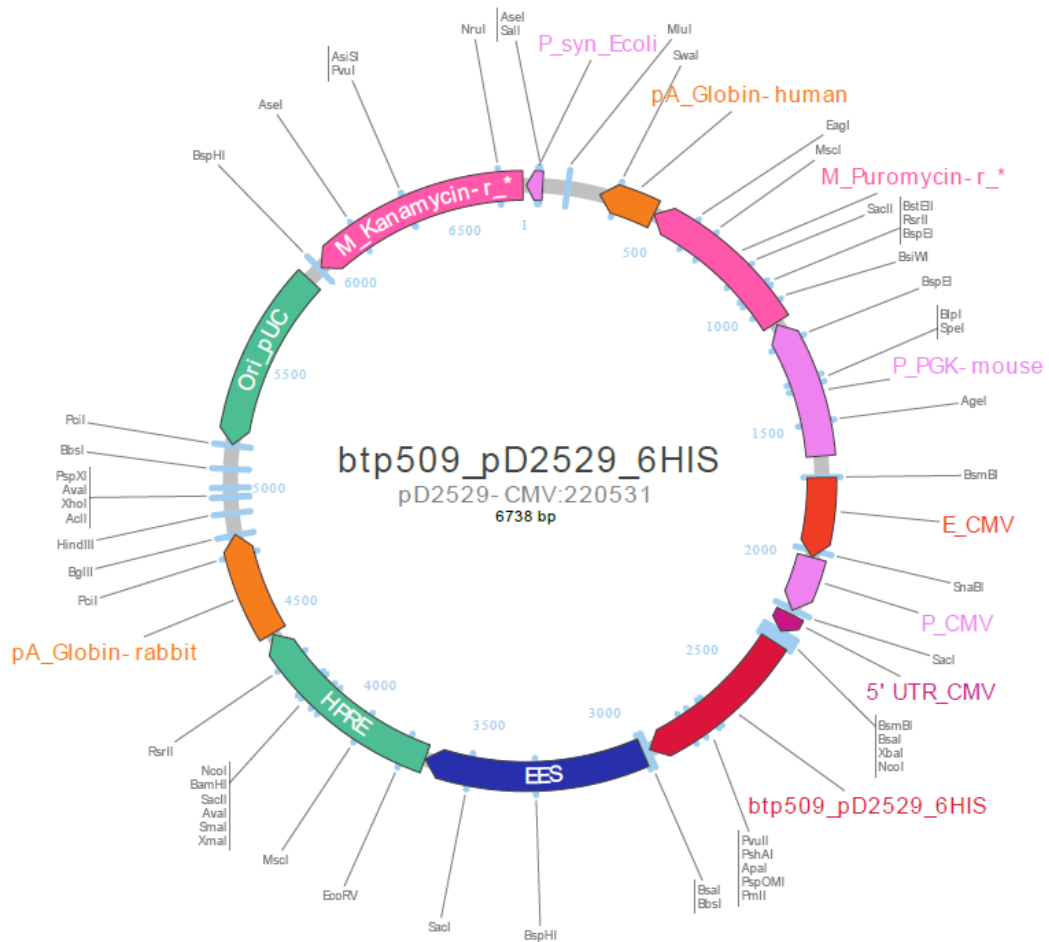

Supplement: Supplemental_Figure_2_ioaf066 [file supplemental_figure_2_ioaf066.pdf]

# IFNT vs PSPB

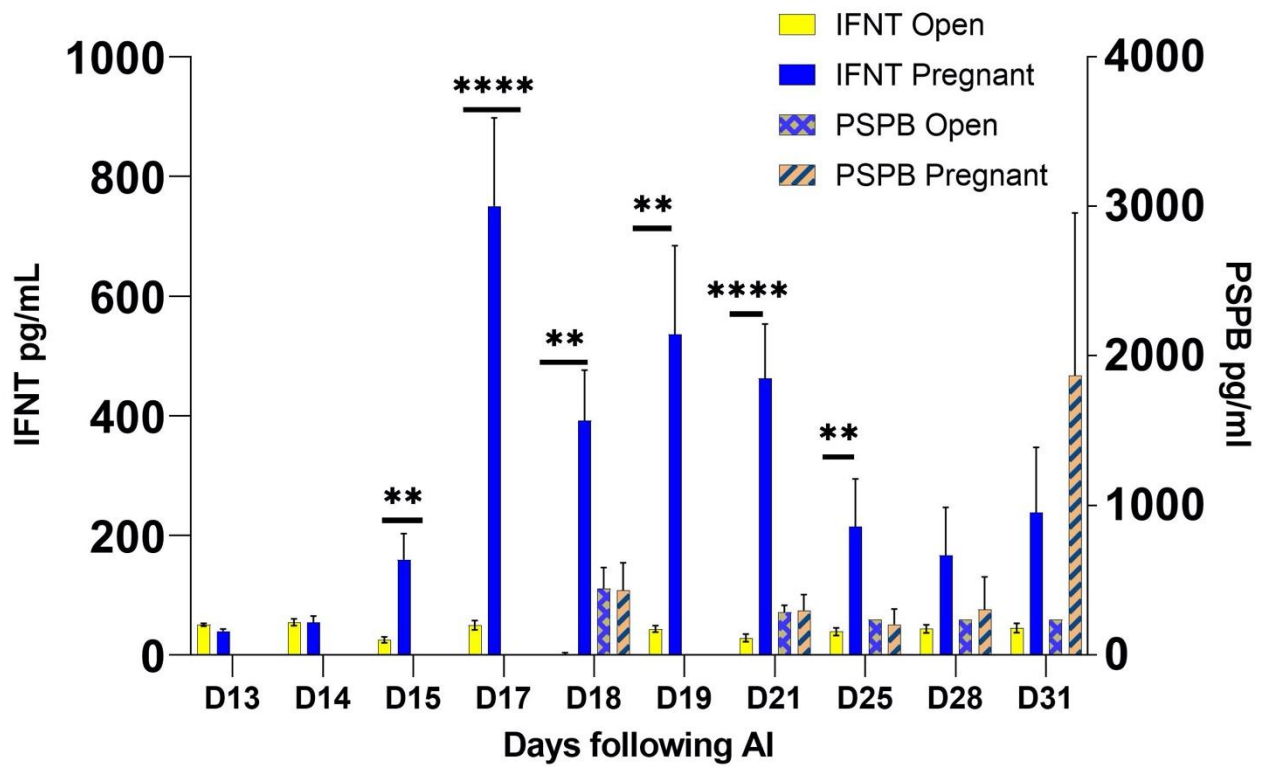

Supplement: Supplemental_Figure_3_ioaf066 [file supplemental_figure_3_ioaf066.pdf]
