# Supplementary material for: Early identification of bovine pregnancy status and embryonic mortality
Source: Biol Reprod. 2025 Mar 28;112(5):981–95. doi: 10.1093/biolre/ioaf066 (PMC12078079; doi:10.1093/biolre/ioaf066)
Supplement: Supplemental_Figure_4_ioaf066 [file supplemental_figure_4_ioaf066.pdf]

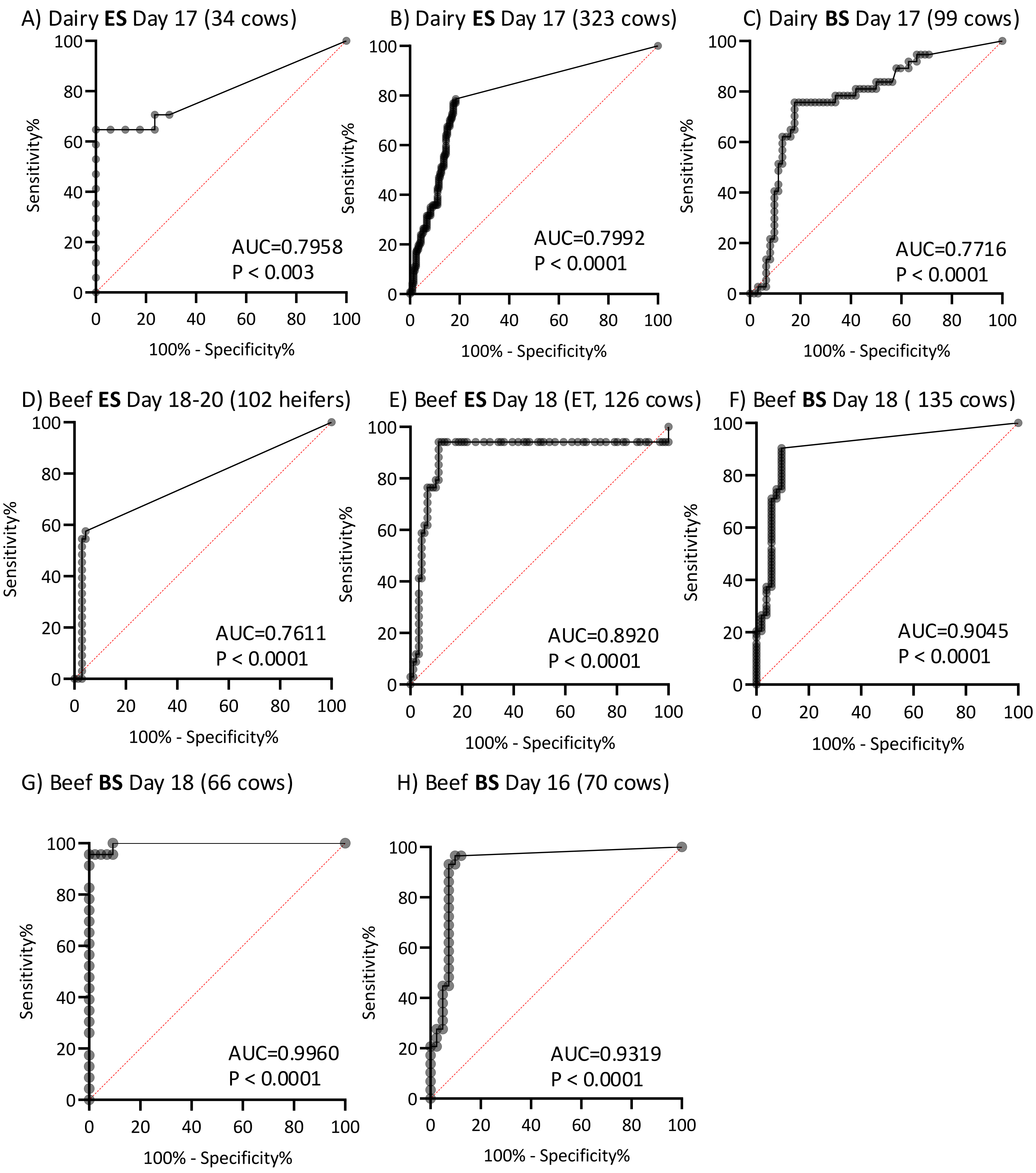
A) Dairy ES Day 17 (34 cows)
AUC=0.7958
P < 0.003
B) Dairy ES Day 17 (323 cows)
AUC=0.7992
P < 0.0001
C) Dairy BS Day 17 (99 cows)
AUC=0.7716
P < 0.0001
D) Beef ES Day 18-20 (102 heifers)
AUC=0.7611
P < 0.0001
E) Beef ES Day 18 (ET, 126 cows)
AUC=0.8920
P < 0.0001
F) Beef BS Day 18 (135 cows)
AUC=0.9045
P < 0.0001
G) Beef BS Day 18 (66 cows)
AUC=0.9960
P < 0.0001
H) Beef BS Day 16 (70 cows)
AUC=0.9319
P < 0.0001
Sensitivity%
100% - Specificity%
