# Supplementary material for: Early identification of bovine pregnancy status and embryonic mortality
Source: Biol Reprod. 2025 Mar 28;112(5):981–95. doi: 10.1093/biolre/ioaf066 (PMC12078079; doi:10.1093/biolre/ioaf066)
Supplement: Supplemental_Table_1_ioaf066 [file supplemental_table_1_ioaf066.docx]

| **Supplemental Table 1**. Calculated parameters of open cow test (OCT) results from Os Cervix and Mid cervix collections based on pregnancy diagnosis by ultrasound in dairy cows after insemination (AI). The study was conducted at Dairy B using the new bovine swab device (Fig. 1). External cervical os samples were collected followed by mid-cervical collections in the same cow. | | | | | |
| --- | --- | --- | --- | --- | --- |
|  |  | Os Cervix (n=99) | | Mid-Cervix (n=99) | |
| Endpoints | Definition | Value | 95% CI | Value | 95% CI |
| TP (n) | P by OCT and US | 35 | - | 35 | - |
| TN (n) | NP by OCT and US | 18 | - | 22 | - |
| FP (n) | P by OCT and NP by US | 44 | - | 40 | - |
| FP (%) | (FP/FP+TN) | 71 | 59.7-82.3 | 64.5 | 52.6-76.4 |
| FN (n) | NP by OCT and P by US | 2 | - | 2 | - |
| FN (%) | (FN/FN+TP) | 5.4 | 0-12.7 | 5.4 | 0-12.7 |
| ACC (%) | (TP+TN/n) | 53.5 | 43.7-63.4 | 57.5 | 47.8-67.3 |
| Se (%) | (TP/TP+FN) | 94.6 | 87.3-1 | 94.5 | 87.3-1 |
| Sp (%) | (TN/TN+FP) | 29 | 17-40.3 | 35.5 | 23.5-47.4 |
| PPV (%) | (TP/TP+FP) | 44 | 33.4-55.3 | 46.6 | 35.4-58 |
| NPV (%) | (TN/TN+FN) | 90 | 76.9-1 | 91.7 | 80.6-1 |
| EEM (%) | (FP/TP+FP+FN) | 54.3 | - | 51.9 | - |
| P=Pregnant, NP= Non-pregnant, TP= True Positive, TN= True Negative, FP= False Positive, FN= False Negative, ACC=Accuracy, Sp=Specificity, Se=Sensitivity, PPV=Positive Predictive Value, NPV=Negative Predictive Value, EEM=Estimated Embryonic Mortality, AI= Artificial Insemination, ES= Equine Swab Device, BS= Bovine Swab Device, US=Ultrasound. | | | | | |
