# Supplementary material for: Early identification of bovine pregnancy status and embryonic mortality
Source: Biol Reprod. 2025 Mar 28;112(5):981–95. doi: 10.1093/biolre/ioaf066 (PMC12078079; doi:10.1093/biolre/ioaf066)
Supplement: Supplementary_Figure_Legends_ioaf066 [file supplementary_figure_legends_ioaf066.docx]

**Supplementary Figure Legends**

**Supplemental Figure 1.** Isolation of the bovine IFNT cDNA clone (A), testing of polyclonal antibodies against rbIFNT using western blot (B) and validation of a 5 h specific and sensitive bIFNT ELISA (C). The cDNA insert was subcloned by a commercial company into an expression vector followed by transfection into HEX cells. The resulting purified rbIFNT was used to generate poly clonal antibodies in goats and rabbits. The most immunogenic anti-rbIFNT antibodies were rabbit 51 and goat 5670 polyclonal antibodies. In B, rbIFNT was loaded at 100 ng/lane and the western blots shown were using Goat 51 antibody at 1:1,000 (75 µg/ml) and rabbit 5670 antibody at 1:10,000 7.5 μg/ml. Secondary polyclonal antibody was either donkey anti-goat (source) or anti-rabbit (source) horse radish peroxidase at 1:2,000.

**Supplemental Figure 2**. Map for the bovine trophoblast protein (BTP) 509 IFNT expression vector.

**Supplemental Figure 3.** IFNT and PSPB concentrations in external os swab (equine device) samples on days 13-31. All samples were tested neat (not diluted). Values represent means ± standard error. There was no significant detection of PSPB when using external cervical os commercial equine cotton swab devices on days tested. IFNT was lower (**, P < 0.01, ** P < 0.001) in open compared to pregnant swab samples on days tested. Pregnancy status was determined by ultrasound on day 32. The IFNT concentrations were qualitative and not adjusted to 0 when values were less than the limit of detection for the assay.

**Supplementary Fig. 4. Receiver Operator Characteristics curves for each study in Table 1 and Table 2.** Note that the relationship between sensitivity and specificity improved when using the bovine swab (BS) compared to the equine swab (ES) device. “Violin” profiles for these studies are plotted in **Fig. 5**.
